# Photoluminescence Detection of Polytype Polarization in r‑MoS_2_ Enabled by Asymmetric Dielectric Environments

**DOI:** 10.1021/acsnano.5c10905

**Published:** 2025-10-02

**Authors:** Idan Kizel, Omri Meron, Dror Hershkovitz, Maayan Vizner Stern, Alon Ron, Moshe Ben Shalom, Haim Suchowski

**Affiliations:** † Condensed Matter Physics Department, School of Physics and Astronomy, Faculty of Exact Sciences, 26745 Tel Aviv University, Tel Aviv 6997801, Israel; ‡ Center for Light-Matter Interaction, 26745 Tel Aviv University, Tel-Aviv 6997801, Israel

**Keywords:** polar van der Waals polytypes, SlideTronics, 2D ferroelectric materials, rhombohedral molybdenum disulfide, photoluminescence

## Abstract

The rhombohedral (r) polytypes of transition metal dichalcogenides (TMDs) constitute a class of 2D ferroelectric materials, where lateral shifts between parallel layers induce reversible out-of-plane polarization. This emerging field, known as *SlideTronics*, holds significant potential for next-generation electronic and optoelectronic applications. Previous research has extensively studied how electrical and chemical doping affects excitonic signatures in conventional 2H-TMDs and how dielectric environments influence their optical properties. However, the impact of intrinsic polarization of these ferroelectric materials in asymmetric dielectric environments remains largely unexplored. Here, we demonstrate a striking polarization-dependent photoluminescence (PL) contrast of up to 400% between ferroelectric domains in bilayer and trilayer rhombohedral molybdenum disulfide (r-MoS_2_). This pronounced contrast arises from an asymmetric dielectric environment, which induces polarization-dependent shifts in the Fermi energy, leading to modulation of the exciton–trion population balance. A detailed temperature-dependent line shape analysis of the PL, conducted from 4 K to room temperature, reveals domain-specific trends that further reinforce the connection between polarization states and excitonic properties. The persistence of these distinct optical signatures at room temperature establishes PL as a robust and noninvasive probe for ferroelectric domain characterization, particularly in fully encapsulated device architectures where conventional techniques, such as Kelvin probe force microscopy, become impractical.

## Graphical abstract

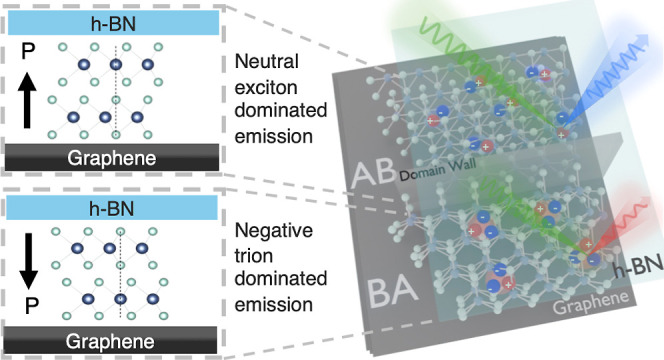

The emergence of sliding two-dimensional (2D) ferroelectric materials,^1,2^ particularly r-TMDs,^3^ has opened a new frontier in quantum materials. In these systems, lateral shifts between crystalline layers can induce reversible changes in out-of-plane electric polarization,^4,5^ enabling applications ranging from nonvolatile memories to high-performance photodetectors.^5–9^ This sliding ferroelectricity in r-MoS_2_ arises from cumulative contributions across interfaces,^3,10^ with a doping-dependent polarization saturation around 10 layers.^11^

Beyond their ferroelectric properties, these materials exhibit remarkable excitonic phenomena, hosting exceptionally stable Coulomb-bound electron–hole pairs that are highly sensitive to their local environment.^12,13^ Recent studies have used reflection contrast as an optical readout method to demonstrate that polarization switching can be achieved via domain wall release in few-layer r-MoS_2_ under symmetric dielectric environments.^6,14^ However, a significant challenge remains: the optical signatures are identical for opposing polarization orientations, necessitating complex electrical measurements to determine the underlying configuration. This limitation becomes particularly acute in device architectures, where the active layer must be fully encapsulated between top and bottom graphene gates for functionality, preventing direct surface potential measurements via conventional techniques like Kelvin probe force microscopy (KPFM).

Photoluminescence (PL) spectroscopy emerges as a promising alternative, offering detailed insights into excitonic phenomena in MoS_2_.^15^ The high sensitivity of PL to doping effects,^16,17^ defects,^18^ and dielectric environments^19^ suggests its potential for probing ferroelectric domains. However, the influence of intrinsic polarization on PL signatures in r-stacked MoS_2_ remains largely unexplored, particularly in asymmetric device geometries relevant for applications.

Here, we demonstrate that distinct stacking configurations (of the same polytype) in bilayer and trilayer r-MoS_2_, when integrated with asymmetric dielectric environments, produce pronounced differences in the PL intensity and spectral features. By engineering this asymmetric screening, we overcome the previous optical degeneracy that prevented a priori characterization of the polytype orientation. The dominant mechanism underlying our observed PL modulation is a doping-induced shift in the neutral exciton-to-trion population balance, which manifests itself in both intensity variations and spectral reshaping. Through temperature-dependent measurements from 4 to 295 K and detailed line shape analysis, including the evolution of spectral features, such as peak positions, line widths, and overall intensity, we show that these optical signatures remain distinguishable at room temperature, establishing a practical, noninvasive technique for mapping ferroelectric domains in device-ready architectures.

## Results and Discussion

Our investigated sample includes a bilayer and trilayer r-MoS_2_ encapsulated between a trilayer graphene substrate and an 8 nm thick hexagonal boron nitride (h-BN) top layer. Bilayer r-MoS_2_ (Figure 1a, left) consists of two distinct stacking configurations that correspond to the same polytype (crystal structure) flipped upside down: AB configuration, where sulfur atoms in the bottom layer are eclipsed by molybdenum atoms in the top layer, resulting in upward intrinsic polarization, and BA configuration, shifted by an additional bond length which is equivalent to a mirror operation over the interface with a downward-facing intrinsic polarization. The trilayer domains (Figure 1a, right) present stacking configurations of two polar interfaces: ABC and CBA, which are equivalent polytypes exhibiting upward and downward polarization, respectively, along with the inequivalent ABA and BAB crystals that both result in zero net out-of-plane polarization.^3^ While wide-field optical microscopy under white light illumination clearly distinguishes between regions of different layer thicknesses (Figure 1b), it fails to detect the spatial distribution of the stacking configurations. To validate our subsequent optical characterization method, we first performed KPFM measurements to map the surface potential distribution over the sample (Figure 1c). As mentioned, although KPFM traditionally serves as an effective tool for probing ferroelectric domains, its application becomes impractical in fully gated devices, where the active layer is encapsulated. Throughout this work, the KPFM map serves as a ground-truth reference for our optical characterization technique. All measurements were conducted using PL spectroscopy with a 532 nm laser excitation under controlled cryogenic conditions, allowing for characterization at temperatures ranging from 4 K to room temperature (see the Methods section for details).

**Figure 1:**
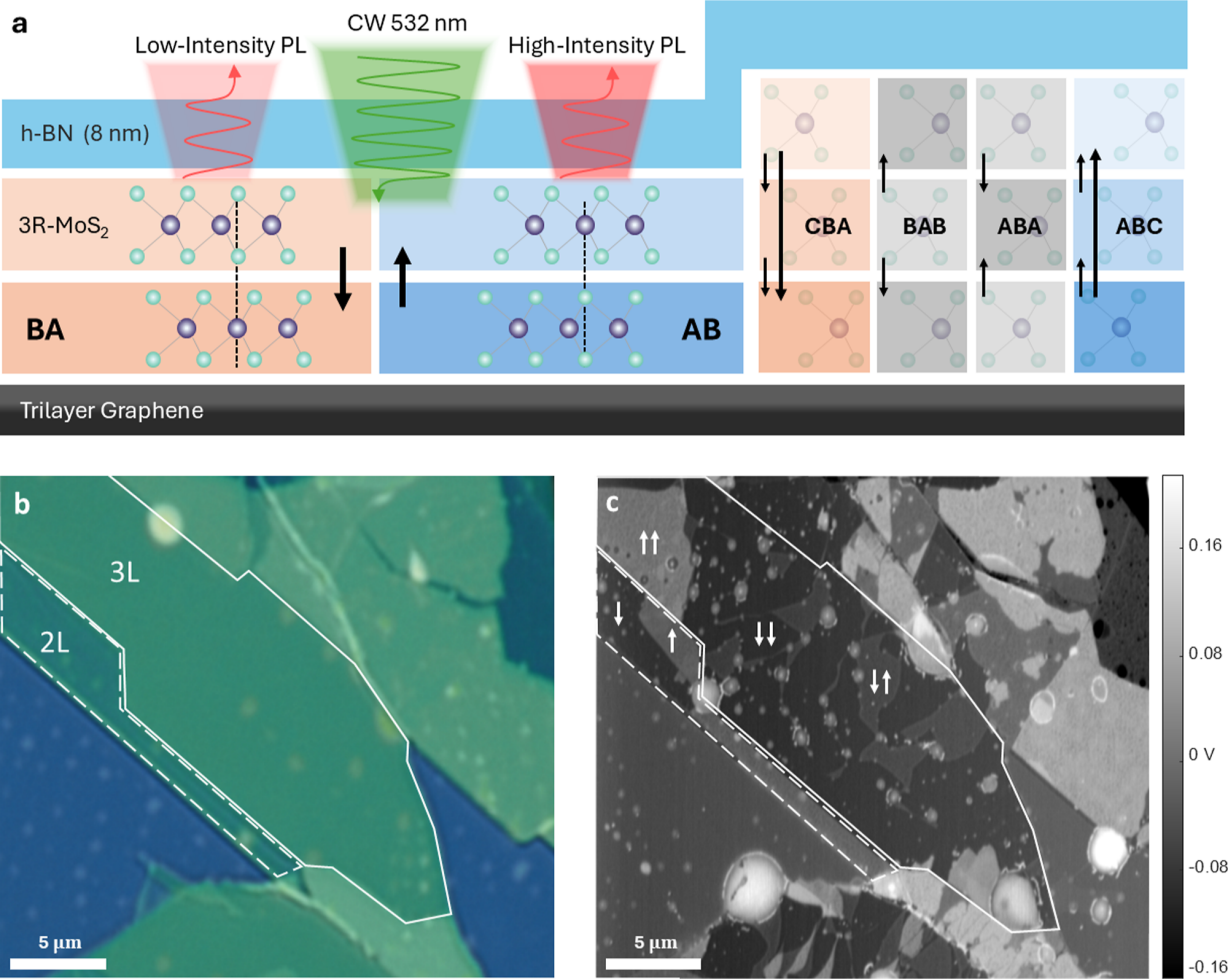
Sample architecture and characterization. (a) Schematic cross-section showing bilayer and trilayer r-MoS_2_ encapsulated between trilayer graphene and h-BN (8 nm). Short (long) black arrows mark interfacial (total) polarization. (b) Optical micrograph showing trilayer (solid white outline) and bilayer (dashed white outline) regions. (c) Kelvin probe force microscopy (KPFM) map revealing ferroelectric domains from different stacking configurations.

The typical PL spectrum of MoS_2_ is composed of distinct excitonic features dominated by the A and B exciton transitions and their corresponding negatively charged trion states. The A exciton (X_A_^0^) typically appears in the range of 1.85–1.95 eV, with its associated trion (X_A_^–^) red-shifted by approximately 20–40 meV.^16,20^ The higher-energy B exciton (X_B_^0^) is observed around 2.00–2.10 eV, along with its trion state (X_B_^–^) at slightly lower energies.^21^ The exact peak positions and line width of these features exhibit notable dependence on temperature,^22–24^ sample quality, and the surrounding dielectric environment.^19^ Additionally, when measuring samples on trilayer graphene using 532 nm (2.33 eV) excitation, two characteristic Raman features are expected to appear within our spectral window: the G peak at approximately 2.08 eV and the 2D peak at around 1.99 eV.^25^

We begin by examining bilayer r-MoS_2_ domains under the cryogenic conditions. Figure 2a presents PL spectra obtained at 4 K comparing regions with AB and BA stacking configurations. Each spectrum represents a spatial average over the corresponding domains marked in Figure 2c, with shaded areas indicating the standard deviation, thereby reflecting spatial inhomogeneities arising from the sample quality. The AB-stacked domains, characterized by upward intrinsic polarization, exhibit markedly enhanced PL intensity, showing approximately 300% stronger emission compared to BA-stacked regions with downward polarization. This pronounced intensity modulation is visualized through the spatially resolved, integrated PL intensity map (Figure 2d), which reveals robust optical contrast between domains of opposing polarization. Direct comparison between the surface potential domains identified via KPFM measurements (Figure 2c) and our PL intensity map demonstrates an unambiguous correlation between the local stacking configuration and emission intensity. The spatial resolution of our PL mapping technique is ∼450 nm, primarily limited by the optical diffraction limit of approximately λ/(2NA), where λ is the excitation wavelength and NA is the focusing objective numerical aperture.

**Figure 2:**
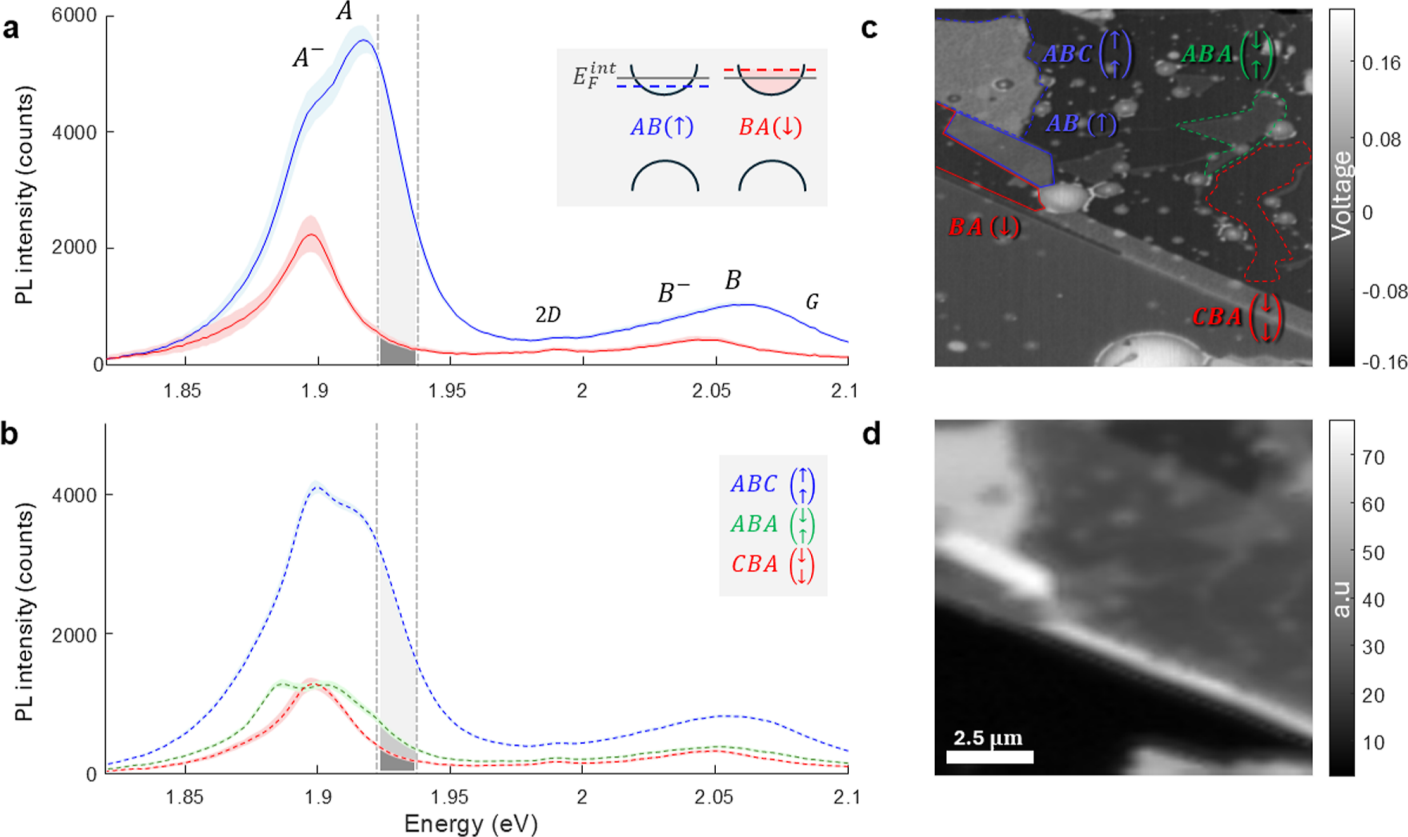
Stacking-dependent PL spectra and domain identification. (a) Low-temperature (4 K) PL spectra from bilayer AB (blue) and BA (red) domains. Shaded areas represent standard deviation across multiple measurements. Gray-shaded range: spectral window for intensity integration in panel (d). Inset: charge transfer-induced Fermi-level shift. (b) Trilayer spectra from ABC (blue), CBA (red), and ABA (green) configurations. (c) KPFM map, serving as ground-truth reference, with colored overlays matching spectra in (a,b). (d) Spatially resolved integrated PL intensity map at 4 K showing one-to-one correspondence with KPFM-identified domains.

A detailed examination of the AB spectrum (Figure 2a) reveals several distinct features: two peaks in the A exciton region at approximately 1.89 eV (attributed to a negative trion) and 1.92 eV (attributed to a neutral exciton) alongside the B exciton region, where we observe a broad peak at around 2.06 eV (also attributed to the corresponding two contributions). In contrast, the BA configuration spectrum exhibits primarily a single peak in the A exciton region, which we attribute to the A negative trion, and another single B exciton peak. Both AB and BA spectra demonstrate an asymmetric tail at low energies. The graphene Raman peaks at 2.08 eV (G) and 1.99 eV (2D) remain visible in both configurations. This stark contrast in spectral features, particularly the enhanced PL in AB domains, can be understood through a charge transfer mechanism between r-MoS_2_ and the graphene substrate driven by intrinsic ferroelectric polarization. To validate the charge transfer mechanism, based on previous studies demonstrating charge transfer between monolayer MoS_2_ and few-layer graphene due to differences in work functions, we performed additional KPFM measurements on a reference sample containing both monolayer and bilayer r-MoS_2_ regions. We calculated absolute work function values using the established literature value for monolayer MoS_2_ (∼4.7 eV).^26,27^ The ∼60 mV potential difference between AB and BA bilayer domains (see Supporting Information Figure S6) corresponds to absolute work functions of ∼4.65 eV and ∼4.71 eV for the AB and BA bottom layers, respectively. Since trilayer graphene has a work function of ∼4.4 eV,^28^ this creates preferential charge transfer from graphene to MoS_2_ in the BA configuration while reducing transfer in the AB configuration (for more details, see Supporting Information Section S9). This difference in charge transfer leads to enhanced electron accumulation in the BA domains, increasing the intrinsic n-type character of MoS_2_ ^29^ and resulting in a trion-dominated photoluminescence. In contrast, the reduced electron density in the AB domains suppresses trion formation and favors neutral exciton emission.^30^ Since the trion PL emission is less affected by such charge transfer effects,^31^ the increased neutral exciton population results in enhanced PL emission. To further support this interpretation, we estimated the electron densities in both domains using the 2D mass action law applied to the trion-to-exciton PL intensity ratio. This model, commonly used in doped MoS_2_ systems,^32,33^ yields values of ∼7 × 10^12^ cm^–2^ in the AB domain to around ∼1.7 × 10^13^ cm^–2^ in the BA domain at room temperature (see Supporting Information Section S6). Although this carrier density difference implies a Fermi-level shift of ∼10 meV, its contribution to the measured ∼10 mV surface potential contrast lies within the measurement error and is negligible compared to the polarization-induced effect (see Supporting Information Section S8). This modulation of the excitonic landscape is enabled by asymmetric stacking of the trilayer graphene substrate relative to the intrinsic polarization direction, highlighting the critical role of the device heterostructure sequence in observing these effects.

Furthermore, while the overall PL response could be influenced by multiple mechanisms, we find that doping-induced changes in the exciton–trion population balance provide the dominant contribution to our observations. The intrinsic polarization in bilayer r-MoS_2_ inherently induces an intralayer exciton splitting of ∼10 meV,^34^ which can be further modulated by the asymmetric dielectric screening environment.^35–37^ Due to the distinct screening properties of graphene and h-BN,^38,39^ this splitting is expected to be enhanced when the out-of-plane polarization direction faces h-BN (AB configuration) and reduced when it faces graphene (BA configuration), as demonstrated by Liang et al.^40^ While our detailed analysis supports the presence of these subtle effects, they remain secondary to the doping-mediated PL modulation (see Supporting Information Section S3 for further details). Focusing on the trilayer configuration, Figure 2b presents the spatially averaged PL spectra for the different trilayer domains. The ABC configuration and ABA/BAB configurations show a significant PL increase of 400% and 150%, respectively, compared to the CBA configuration. There is no observable difference between the neutral configurations (ABA and BAB), and it is unclear if both exist in our sample since KPFM mapping cannot differentiate between these zero polarization states. Interestingly, the CBA stacking, which has a net downward polarization and is expected to display an enhanced n-type character and a trion-dominated spectrum, emits less radiation than the ABA/BAB domain; the latter maintains a similar exciton–trion population ratio typical of naturally n-doped MoS_2_. These findings from the trilayer case reaffirm our hypothesis regarding the bilayer configuration: upward polarization is linked with a higher density of neutral excitons compared to the zero net polarization scenario, leading to a significant PL enhancement, whereas downward polarization increases the trion population but results in only a slight decrease in PL emission.

The appearance of two distinct peaks in our neutral polarization configuration reveals intriguing insights about the stacking order when compared with the recently reported symmetrically h-BN-encapsulated case.^14^ In that study, the ABA configuration exhibits two peaks: one from the distinct middle layer exciton and another from the merged energetically degenerate top and bottom layers, while their BAB configuration showed a single merged peak due to the energetic alignment of all three layers. In our graphene-MoS_2_-hBN structure, despite the highly asymmetric dielectric environment, we expect similar fundamental behavior but with modified energies. Specifically, in the ABA configuration, the three distinct excitonic states are shifted by the asymmetric screening: the bottom layer experiences strong screening from the semimetallic graphene substrate, the middle layer is sandwiched between MoS_2_ layers, and the top layer faces the weaker screening h-BN. Unlike the symmetric dielectric case where the intralayer excitons of top and bottom layers are degenerate, in our asymmetric environment, each intralayer exciton experiences different screening, resulting in three distinct exciton peaks. However, our measurements show only two resolved peaks because the middle- and top-layer intralayer excitons energies are too close to be resolved separately at 4 K. The lower-energy peak originates from the graphene-screened bottom layer, while the higher-energy peak contains the merged middle- and top-layer signals, with a hint of their separation visible at 1.92 eV. Conversely, in the BAB configuration, despite the asymmetric screening environments, we would expect a single merged peak, as the inherent energy-level alignment of the three layers in this stacking persists, similar to the symmetrically encapsulated case. Therefore, our observation of two distinct peaks with a separation of approximately 34 meV, and the hint for another peak (with smaller separation), strongly suggests an ABA configuration in our sample.

Temperature-dependent PL measurements provide further insights into the stability and evolution of the stacking-dependent optical response. Within the bilayer regions, we observe systematic changes in the PL spectra with an increase in temperature, as shown for AB-stacked domains (Figure 3a) and BA-stacked domains (Figure 3b). Throughout the measured temperature range, AB-stacked domains maintain stronger emission than BA-stacked domains, though this intensity contrast gradually diminishes toward higher temperatures. Similar temperature-dependent behavior manifests itself in trilayer regions, where the ABC configuration (Figure 3d) maintains enhanced emission relative to both the ABA/BAB (Figure 3e) and CBA (Figure 3f) stackings. Remarkably, the PL intensity contrast between different domains persists up to room temperature, as evidenced by the spatially resolved room-temperature integrated PL map in Figure 3c. This PL map again shows a one-to-one correspondence with the KPFM-identified domains, expanding the PL mapping method throughout all temperature ranges.

**Figure 3:**
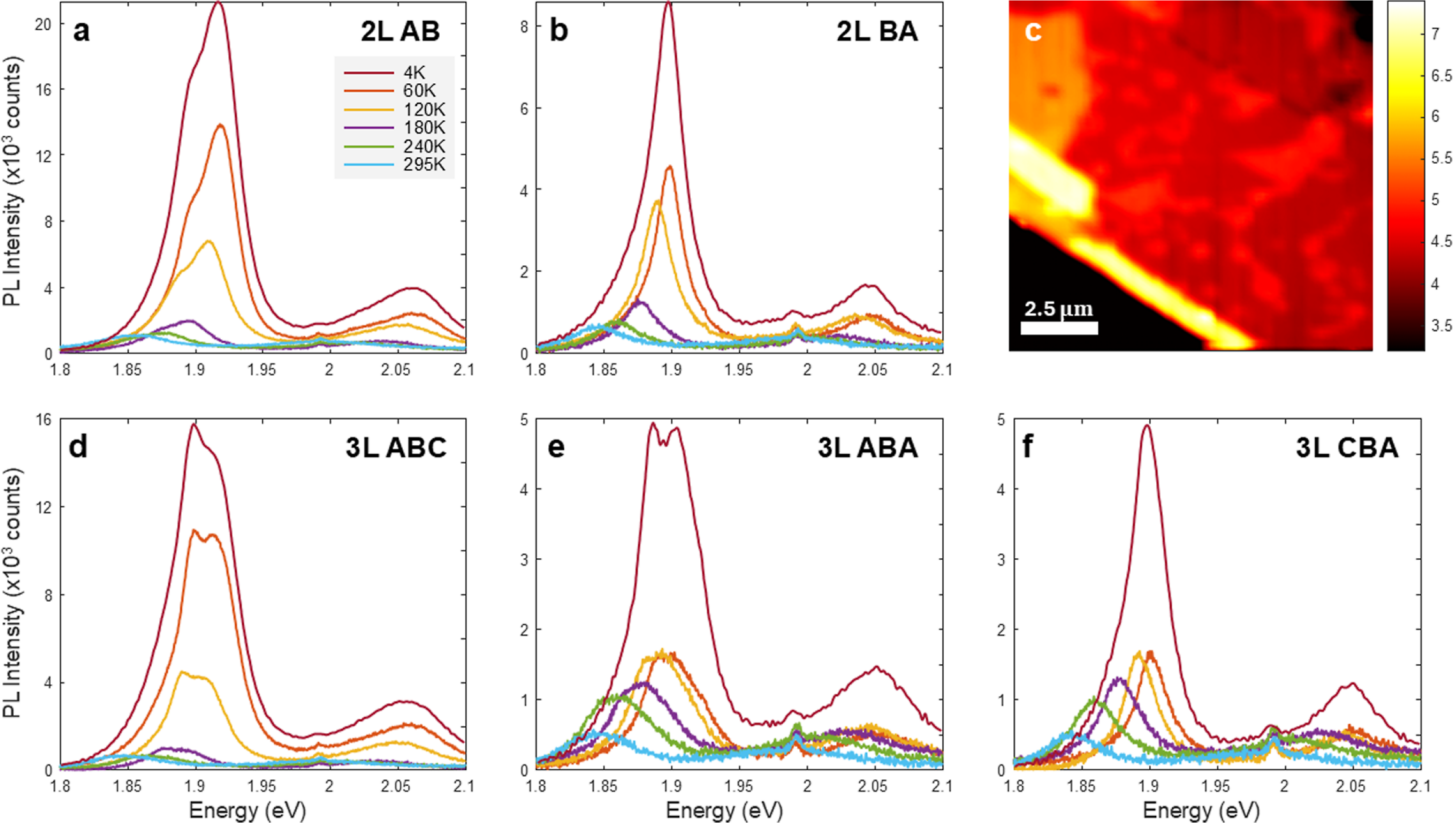
Temperature-dependent PL evolution along stacking configurations, from 4 to 295 K. (a) Bilayer AB stacking, (b) bilayer BA stacking, (d) trilayer ABC stacking, (e) trilayer ABA/BAB configuration, and (f) trilayer CBA stacking. The spectra reveal systematic thermal broadening of excitonic features and a characteristic redshift with increasing temperature while maintaining the relative intensity differences between stacking configurations. (c) Room-temperature (295 K) integrated PL intensity map demonstrating that the stacking-dependent contrast persists even at elevated temperatures, enabling practical domain identification under ambient conditions.

To systematically analyze the distinct excitonic spectral line shapes of the bilayer domains and their temperature-dependent trends, we employed a multipeak fitting procedure incorporating Voigt profiles for each excitonic transition (see Figure 4a,b for 4 K and Figure S3 in Supporting Information for the remaining temperatures). The Lorentzian line width component γ accounts for broadening due to the dephasing and finite lifetime of the excited states, while the Gaussian line width component σ reflects spatial inhomogeneities over the domain arising from local variations in dielectric environment and strain.^41^ For trion peaks, we modified the standard Voigt profile by convolving it with an asymmetric tail function following Christopher et al., accounting for the radiative decay of nonzero momentum trions.^24^ This modification is physically motivated by the unique decay mechanism of trions: unlike excitons, which can decay only radiatively when their momentum lies within the light cone, trions can decay from any momentum state by ejecting an electron that carries away the excess momentum. The exceptional quality of fit achieved by using this modified profile, incorporating the characteristic asymmetric tail, provides additional confidence in our identification and analysis of the trion states.

**Figure 4:**
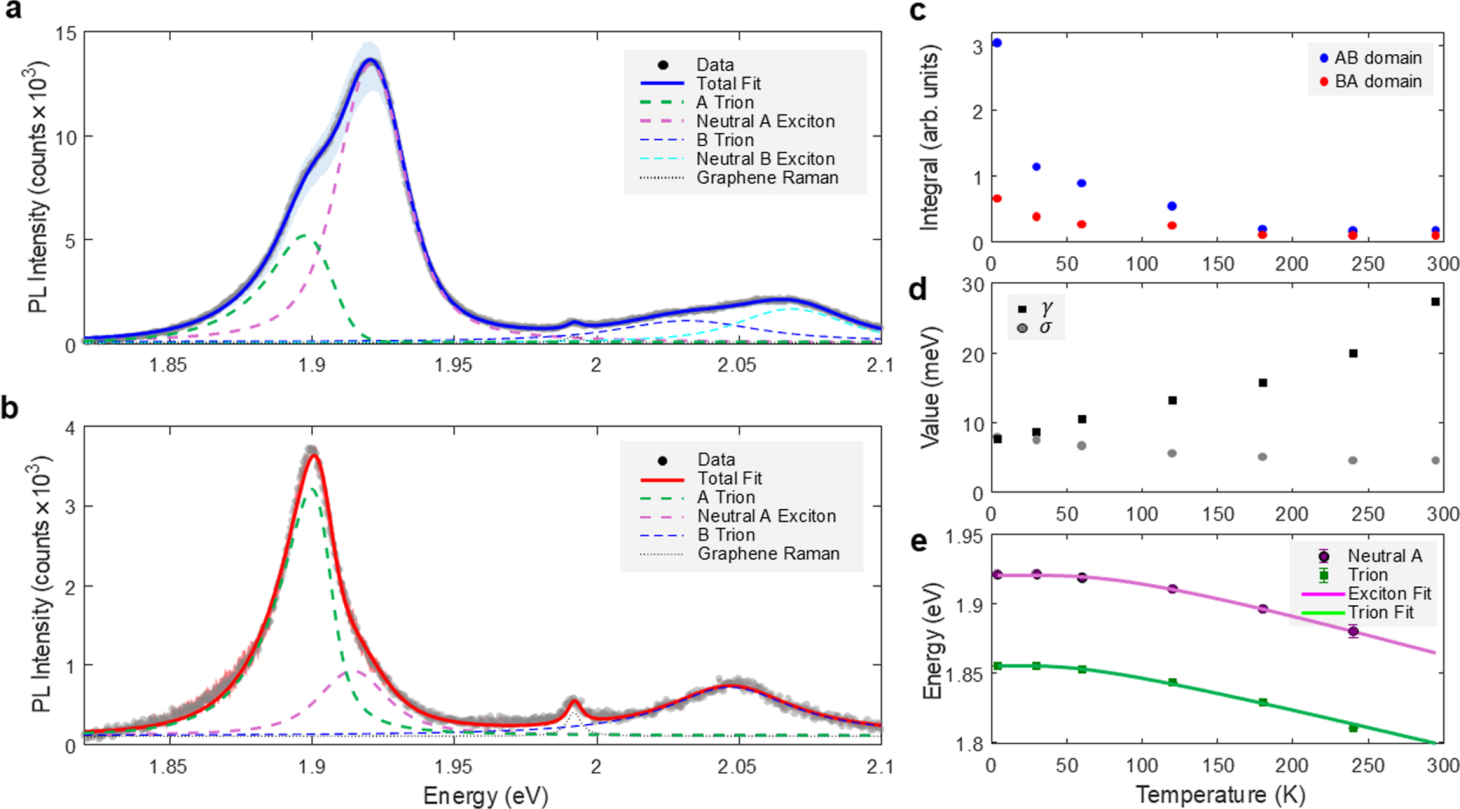
Spectral fitting and temperature-dependent analysis. (a) PL spectrum from the bilayer AB domain at 4 K with multipeak Voigt profile fitting (solid blue) showing A-exciton (purple), A-trion (green), B-trion (blue), and B-exciton (light blue). (b) Corresponding spectrum and fit (red) for the bilayer BA domain. (c) Temperature dependence of integrated PL intensity for AB and BA configurations. (d) Evolution of γ (Lorentzian) and σ (Gaussian) line width parameters. (e) Temperature evolution of exciton and trion energies (AB configuration) with fits to the O’Donnell–Chen model.

We evaluated three essential parameters over the entire temperature range to capture the main temperature-dependent trends. The first parameter is the integrated PL intensity presented in Figure 4c. Though the PL intensity in the AB-stacked domains is consistently stronger than that in the BA-stacked domains over the entire temperature range, the disparity in intensity between these stacking types decreases as the temperature rises. This reduction in PL emission with increasing temperature can be attributed to two main effects: increased thermal population of states away from the *K* point and enhanced phonon scattering, both of which reduce the probability of direct exciton transitions. Notably, the trion emission shows greater resilience to this thermal reduction compared to neutral excitons (see Supporting Information, Figure S3). This enhanced stability arises from the trion’s additional charge carrier, which helps maintain momentum conservation during the recombination process even when scattered to states beyond the light cone and away from the *K* point, unlike neutral excitons, which require strict momentum matching. The second parameter group we analyze, the excitonic peak line widths, generally shows a systematic broadening with increasing temperature, following the expected behavior for increasing phonon-induced dephasing. This broadening effect is attributed to the line width γ of the Lorentzian component, while the spatial inhomogeneous broadening parameter σ remains largely temperature-independent (see Figure 4d). Lastly, the third parameter we analyze is the excitonic energy redshift with increasing temperature. This redshift can be mainly attributed to bandgap renormalization, which is commonly described by the O’Donnell–Chen semiphenomenological model.^42^ Figure 4e, which presents the exciton and trion energy as a function of temperature for the bilayer AB-stacked domain, demonstrates a good fit to the semiphenomenological model (see Supporting Information, Section S5, for more details).

## Conclusion

To conclude, we have demonstrated that the interplay between stacking configurations and asymmetric dielectric environments in few-layer r-MoS_2_ produces distinct photoluminescence signatures that enable noninvasive identification of ferroelectric domains. Our systematic investigation revealed several key findings: First, in bilayer r-MoS_2_ at 4 K, upward-polarized (AB) domains exhibit approximately 300% stronger PL emission compared with downward-polarized (BA) domains, with clear spectral differences in the neutral exciton and trion features. This enhancement primarily stems from polarization-induced modulation of the exciton–trion population balance, where upward polarization effectively counteracts the intrinsic n-type doping of MoS_2_. Second, in trilayer domains, we observed a similar pattern where ABC stacking (upward polarization) shows significantly enhanced emission compared with both CBA (downward) and ABA/BAB (neutral) configurations, with spectral features providing insights into the complex interplay between stacking order and excitonic effects.

Our temperature-dependent studies, spanning from 4 K to room temperature, revealed that while thermal effects lead to expected peak broadening and redshifts, the crucial contrast in PL intensity between different stacking configurations persists up to room temperature. Our findings highlight that the asymmetric dielectric environment plays a dual role. First, it can modulate the subtle intralayer excitonic splitting, though this effect is observable only at ultralow temperatures. More importantly, the distinct screening properties of graphene and h-BN create an environment in which the intrinsic polarization can effectively tune the local carrier density. When domains of opposing polarization interact with this asymmetric environment, they experience differential doping effects. These doping effects substantially modify the exciton–trion population balance, resulting in strong PL contrast between domains that persists up to room temperature. This remarkable feature makes our technique particularly valuable for practical applications, as it enables domain identification under ambient conditions without the need for specialized cryogenic instrumentation.

The methodology we propose here addresses a significant challenge in the field: the need for noninvasive characterization of polytype polarization in fully encapsulated device architectures where conventional probing techniques like KPFM become impractical. By leveraging the asymmetric screening effects of trilayer graphene and h-BN in conjunction with the intrinsic polarization of r-MoS_2_, we have established a robust optical approach for mapping polytype polarization domains. These findings not only advance our fundamental understanding of polarization-dependent excitonic phenomena in 2D ferroelectric materials but also provide a practical pathway for developing polarization-sensitive optoelectronic devices that harness the unique properties of r-stacked TMDs.

## Methods

### Device Fabrication

MoS_2_ flakes of various thicknesses were exfoliated from a bulk 3R crystal, obtained from “HQ Graphene”, onto polydimethylsiloxane (PDMS) substrates. Trilayer graphene flakes were exfoliated from a graphite bulk, obtained from “Manchester nanomaterials”, onto SiO_2_ substrates. The MoS_2_ flakes were stacked onto the trilayer graphene substrate at room temperature. h-BN flakes were exfoliated onto a SiO_2_ substrate, and a PDMS/poly­(methyl methacrylate) stamp was used to pick up the h-BN flake and transfer it onto the MoS_2_/graphene stack.

### AFM Measurements

Topography and sideband KPFM measurements were performed using a Park Systems NX10 AFM, employing PointProbe Plus-Electrostatic Force Microscopy (PPP-EFM) n-doped tips with a conductive coating. The mechanical resonance frequency of the tip was 75 kHz, its force constant was 3 N/m, and the cantilever was oscillated mechanically with an amplitude of ≈20 *nm*. We excited the cantilever with an AC voltage amplitude of 2 V and a frequency of 2 kHz. The topography and KPFM signals were obtained separately using a two-pass measurement. The first pass recorded the topography in noncontact mode. In the second pass, the KPFM potential was recorded after lifting the tip an extra 5 nm and following the same topography line-scan, ensuring separation of the topography and the electrical signals. The images in Figures 1c and 2c were acquired using Park SmartScan software, and the data analysis was performed with the Gwyddion program. The stacking order of r-MoS_2_ domains (AB, BA, ABC, CBA, etc.) was determined through a systematic analysis of the KPFM measurements. Following the methodology established in our previous work on polar van der Waals heterostructures,^2^ we identify stacking configurations based on their characteristic surface potential signatures (for more details, see Supporting Information Section 7).

### Optical Characterization

PL spectra were measured using a self-built reflection cryogenic microscope (attoDRY800 with an external objective, LUCPLFLN40X/0.6 NA). Measurements were performed with a linearly polarized 532 nm CW laser using an average power of 250 μW. The PL spectra were measured using a Shamrock 303i spectrometer coupled with an iDus401 CCD camera (sensor cooled via thermoelectric cooling to 205 K). Optical images of the devices were obtained by using a metallurgical microscope. The room-temperature PL map (Figure 3c) was obtained using WITec alpha 300, with a linearly polarized 532 nm CW excitation laser and a 0.95 NA objective.

## Supplementary Material



## Notes

Additional experimental details and methods, device fabrication and characterization, AFM topography and sample micrographs, temperature-dependent PL spectra and multipeak fitting workflow with parameters, model equations and fitted values (O’Donnell–Chen temperature shift, carrier-density extraction from trion/exciton ratios), KPFM methodology, surface potential profiles, and work function analysis (PDF)
