# Supplementary material for: Photoluminescence Detection of Polytype Polarization in r‑MoS2 Enabled by Asymmetric Dielectric Environments
Source: ACS Nano. 2025 Oct 2;19(40):35629–37. doi: 10.1021/acsnano.5c10905 (PMC12530055; doi:10.1021/acsnano.5c10905)
Supplement: Supplementary file 1 [file nn5c10905_si_001.pdf]

Supporting Information for –

Photoluminescence Detection of Polytype Polarization in r-MoS_2 Enabled by Asymmetric Dielectric Environments

Idan Kizel[1,2], Omri Meron[1,2] , Dror Hershkovitz[1,2], Maayan Vizner Stern[1], Alon Ron[1,2], Moshe Ben Shalom[1] and Haim Suchowski[1,2†]

[1]Condensed Matter Physics Department, School of Physics and Astronomy, Faculty of Exact Sciences, Tel Aviv University, Tel-Aviv, 6997801, Israel.
[2]Center for Light-Matter Interaction, Tel Aviv University, Tel-Aviv, 6997801, Israel

Table of Contents

S1 Sample microscope image 2
S2 Sample topography AFM 2
S3 Multi-peak fitting procedure 3
S3.1 Multiple Physical Mechanisms Contributing to the PL Response 4
S4 Differential reflection 5
S5 O'Donnell-Chen model 5
S6 Carrier Density Extraction from Trion-to-Exciton Ratios 6
S7 Stacking Order Determination by KPFM 7
S8 Comparison between interfacial Polarization and Carrier density Contributions to Surface Potential 7
S9 Quantitative Work Function Analysis 8
S10 References 9

S1 Sample microscope image

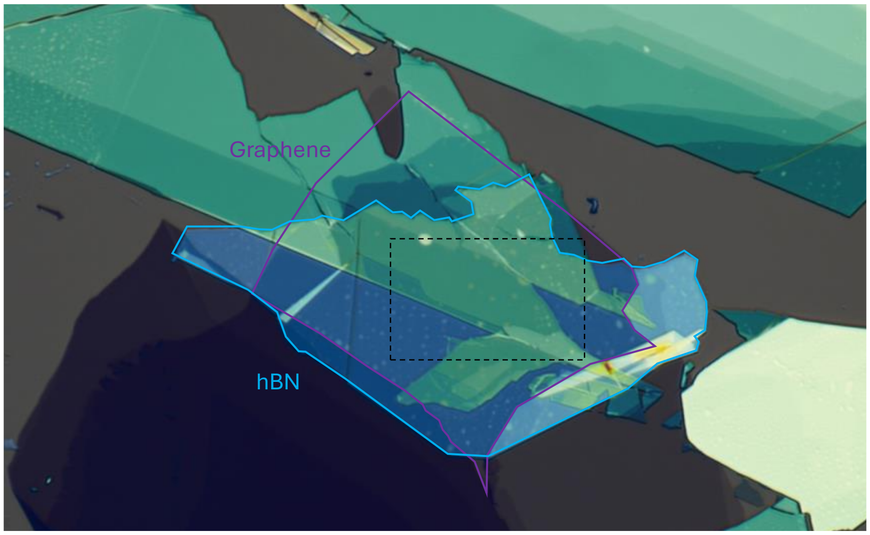

Figure S1: Microscope image of the sample - the 3R-MoS_2 is placed in between a bottom Trilayer Graphene (purple) and top 8 nm thick hBN layer.

S2 Sample topography AFM

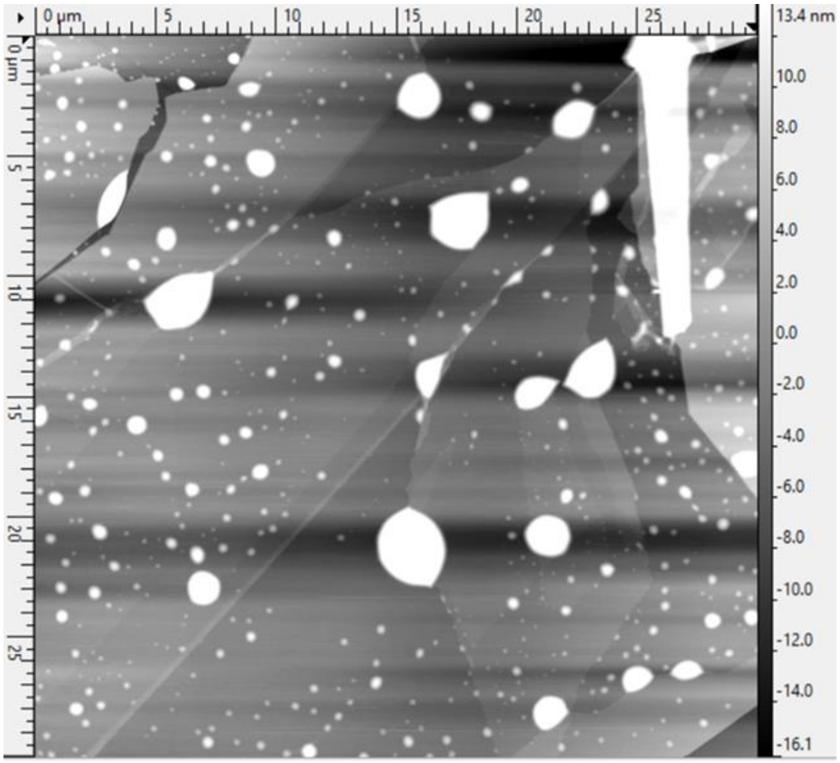

Figure S2: AFM scan of the sample.

S3 Multi-peak fitting procedure

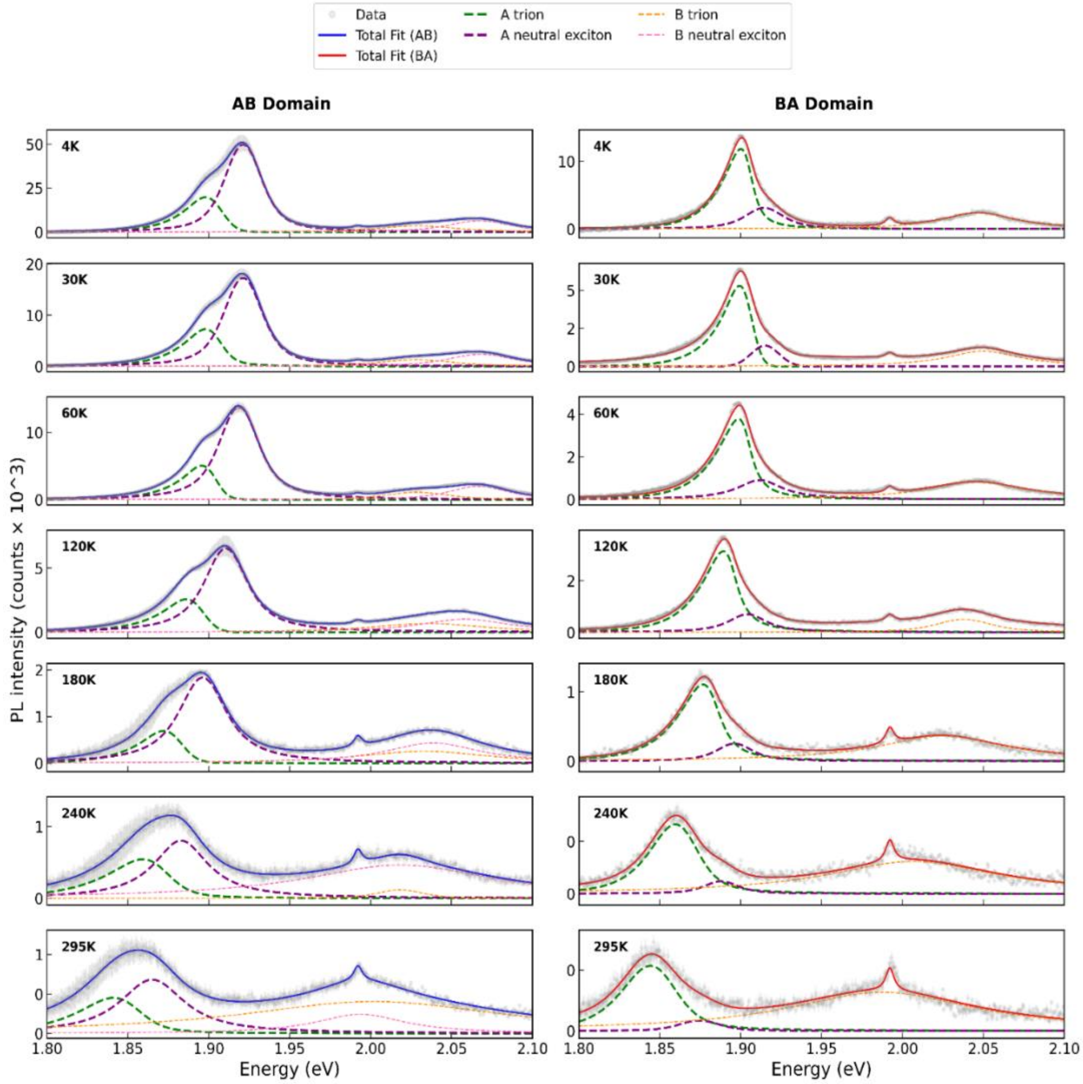

Figure S3: PL Line-shape analysis of bilayer 3R–MoS_2 for AB (left column) and BA (right column) domains across a broad temperature range (4-295 K).

In each panel of figure S3, the measured PL data (gray circles) are fitted to a sum of Voigt-type peaks for the A and B neutral excitons (purple and pink dashed lines), B trions (orange dashed lines), and a Voigt function convolved with a tail for the A trions (green dashed lines). The total fit for the AB domain (blue solid line) and BA domain (red solid line) closely matches the experimental data, illustrating the temperature-dependent evolution of both neutral and charged excitonic resonances.

S3.1 Multiple Physical Mechanisms Contributing to the PL Response

While our main text focuses on the dominant doping-induced effects, the complete PL response in our system involves several simultaneous mechanisms that warrant detailed discussion.

Previous studies have reported that asymmetric interlayer coupling in bilayer 3R-MoS_2 induces an energy offset of approximately 10 meV between intralayer excitons, with the polarization direction pointing toward the intralayer exciton of higher energy (ref [34]). In our specific sample architecture, this intrinsic splitting could be further modulated by the asymmetric dielectric environment, as screening from different substrates has been shown to modify exciton binding energies and optical band gaps in few-layer MoS_2 and other TMDs (ref [35-37]).

The distinct screening properties of graphene versus h-BN create different influences on the intralayer exciton energies (ref [38-39]). In the AB configuration, where the higher-energy layer faces the weakly screening h-BN, one would expect enhanced energy separation between layers. Conversely, in the BA configuration, where the higher-energy layer faces the strongly screening graphene trilayer, this energy separation should be reduced.

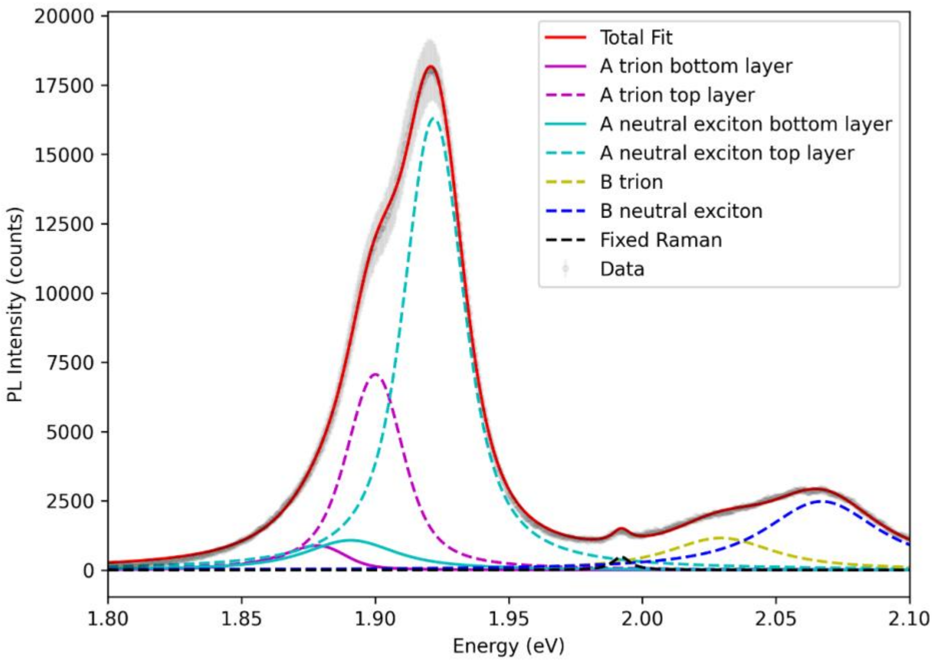

Figure S4: Detailed multi-peak fitting of the low-temperature (4K) PL spectrum from bilayer 3R-MoS_2, capturing the possibility of subtle intralayer exciton and trion splitting. The measured data (gray) is fitted by multiple Voigt profiles (colored curves), with their sum shown as the solid red line. Each excitonic feature is further split to represent top- and bottom-layer contributions influenced by asymmetric dielectric screening and interlayer coupling. Although the main text employs a simpler model of single neutral exciton and trion peaks for clarity, this more detailed approach demonstrates that fine-structure splitting can be robustly incorporated into the analysis while achieving similarly high-quality fits.

Our detailed multi-peak fitting analysis, which includes potential intralayer splitting of both neutral excitons and trions, yields excellent agreement with the experimental data (Figure S4). However, the large number of fitting parameters required and the presence of several broadening effects even at 4K make it challenging to definitively quantify these splitting effects. While these additional mechanisms introduce subtle spectral modifications, the dominant features and intensity modulation in our measurements are most readily explained by doping-induced changes in the exciton-trion population balance.

S4 Differential reflection

To demonstrate the superiority of PL for mapping 3R-MoS_2 domains, we conducted comparative reflectivity measurements using a supercontinuum laser (Fianium Whitelase). The reflectivity intensity map shown in Figure S5 exhibits significantly lower domain contrast compared to the spatially-resolved integrated PL intensity map presented in Figure 2.d of the main text. While technical factors such as white light focusing limitations and laser power fluctuations contribute to this difference, the primary reason stems from the reduced distinction between spectral lineshapes observed in differential reflectivity (dR) measurements compared to PL measurements, as illustrated in Figures S5.b and S5.c. The differential reflectivity was extracted with respect to the graphene substrate covered by the encapsulating hBN layer: $dR = \frac{R_{domain} - R_{substrate}}{R_{substrate}}$

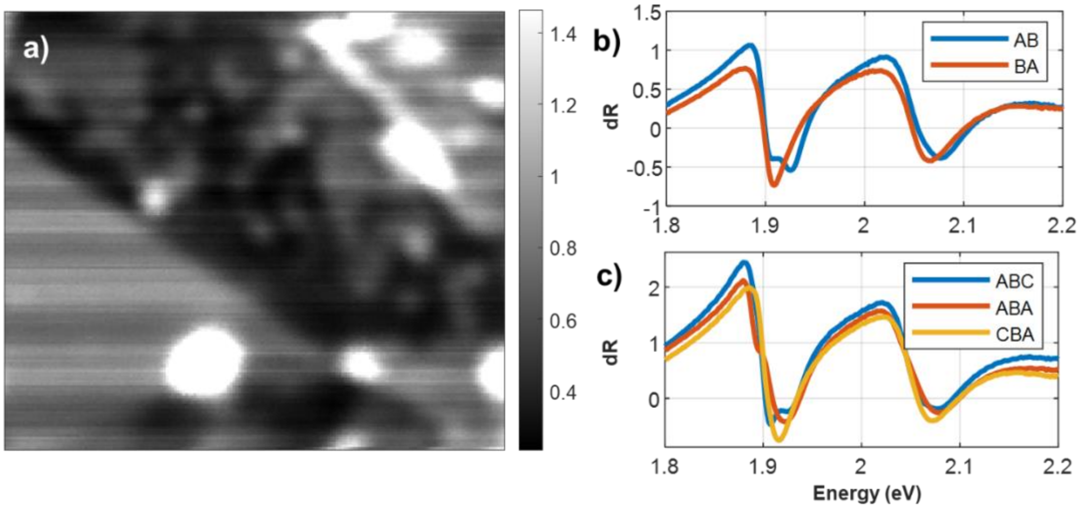

Figure S5: Reflectivity measurements at 4 K. (a) Spatially resolved integrated reflection map (1.910- 1.913 eV). (b-c) Reflection contrast spectrum of (b) bilayer and (c) trilayer domains.

S5 O'Donnell-Chen model

The temperature dependence of the peak positions was analyzed using the O'Donnell-Chen model (ref [42]):

$$\text{(S1)} \qquad E(T) = E_0 - S\hbar\omega\left[\coth\left(\frac{\hbar\omega}{2k_BT}\right) - 1\right]$$

where E_0 is the transition energy at 0 K, S is a dimensionless coupling constant, and $\hbar\omega$ represents the average phonon energy. The model provides good fits for the observed exciton and trion features across our entire temperature range (Figure 4.e in the main text), yielding distinct parameters for different excitonic species. For the neutral A exciton in AB-stacked domains, we obtained:

Table S1: Fitted parameter values from Eq. S1 for different excitonic species in bilayer AB domain

Excitonic Species (AB domain)	E_0 (meV)	S	$\hbar\omega$ (meV)
A exciton	1.921 ± 0.001	1.80 ± 0.06	0.0228 ± 0.0013
A^- trion	1.906 ± 0.014	1.55 ± 0.12	0.0163 ± 0.0034

S6 Carrier Density Extraction from Trion-to-Exciton Ratios

We estimated the carrier density using the standard 2D mass action model for exciton-trion equilibrium, as developed for monolayer MoS_2 by Mouri et al. (ref [16]) and later applied to few-layer systems by Golovynskyi et al. (ref [32-33]). The electron density is calculated via:

$$\text{(S2)} \qquad n_e = \frac{I_{A^-}}{I_A}\frac{\gamma_{ex}}{\gamma_{tr}}\frac{4k_BT}{\pi\hbar^2}\frac{M_A m_e}{M_A^-}\exp\left(-\frac{E_b}{k_BT}\right)$$

where I_{A^-} and I_A are the integrated PL intensities of the neutral exciton and trion emission, respectively. γ_{ex} and γ_{tr} are the radiative decay rates of the neutral excitons and trions, respectively. M_A, M_{A^-} and m_e are the effective masses of the neutral exciton, trion and electron, respectively, and E_b is the trion binding energy. We note that while this formula was originally developed for monolayer MoS_2, Golovynskyi et al. [ref 33] demonstrated its applicability to bilayer systems with appropriate considerations. Our bilayer r-MoS_2 exhibits different stacking from the conventional 2H polytype, leading to different parameters that need to be determined in future studies. Nevertheless, we applied this model as a rough approximation using the parameters described below. Since our sample is a bilayer with an indirect bandgap and a Q-valley CBM, we used complex effective mass terms: $M_A = m_e^K + m_h$ and $M_{A^-} = m_e^K + m_e^Q + m_h$, to account for the assumption that the trion's extra electron, which can carry the excess momentum, resides at the Q valley. We used $m_e^K = 0.35m_0$,$m_h = 0.45m_0$ from Mak et al. (ref [31]), and estimated $m_e^Q = 0.6m_0$ following the theoretical work by Cheiwchanchamnangij and Walter (ref [S1]) which provides a range of $0.55 - 0.82\ m_0$. We used the same γ's ratio as Mouri at al. since we found no other values in the literature.

Using this approach, we obtained electron densities of $6.89 \times 10^{12}\ cm^{-2}$ in the AB domain and $1.7 \times 10^{13}\ cm^{-2}$ in the BA domain. These values fall within the expected range for van

der Waals systems with proximity doping effects and align well with the observed spectral signatures - the lower carrier density in the AB domain correlates with its exciton-dominated emission, while the higher density in the BA domain explains its prominent trion features.

S7 Stacking Order Determination by KPFM

The identification of stacking orders in rhombohedral MoS_2 domains was performed using Kelvin Probe Force Microscopy (KPFM), following established protocols for polar van der Waals structures (see Vizner Stern & Ben Shalom, ref [2]). Figure S6.b presents the KPFM map of a representative sample, where distinct domains are clearly visible. By extracting the surface potential along a marked line across the domain boundary (Fig. S6.a), we resolve a sharp potential step of approximately 60 mV between adjacent domains. This step is consistent with the expected surface potential contrast between AB and BA stackings, corresponding to a single interfacial polarization flip.

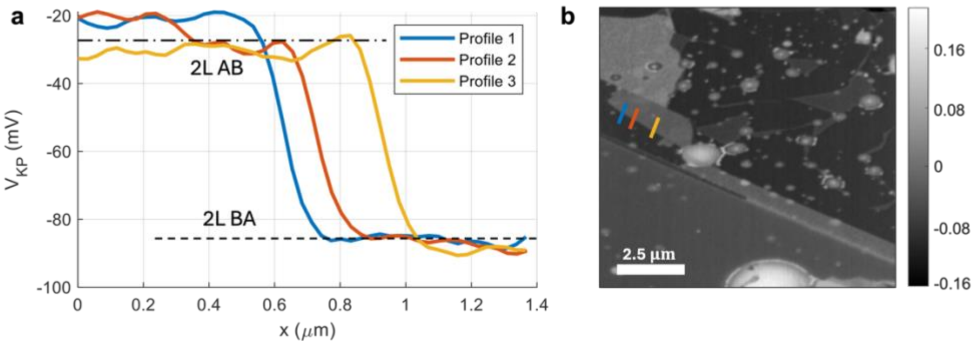

*Figure S6: **Stacking order identification via surface potential mapping. a)** KPFM map of our r-MoS_2 sample showing distinct 2L domains. **b)** Surface potential profiles across domain boundaries reveal a ~60 mV step, consistent with a single interfacial polarization flip between AB and BA stackings.*

S8 Comparison between interfacial Polarization and Carrier density Contributions to Surface Potential

To assess the influence of doping on the surface potential contrast between AB and BA domains, we calculated the Fermi energy shift ΔE_F expected from the extracted carrier density difference ($\Delta n_e \sim 1 \times 10^{13} cm^{-2}$). Using a 2D electron gas approximation with spin and valley degeneracy $g = 4$ and an effective mass $m^* = 0.6\, m_0$ (appropriate for the Q-valley conduction band in r-MoS_2), we find:

$$\Delta E_F = \frac{\pi \hbar^2 \Delta n_e}{g m^*} \sim 10\, meV$$

This energy difference corresponds to a surface potential variation on the order of 10 mV, which is within the uncertainty of our KPFM measurements (±10 mV). In contrast, the measured KPFM potential step between AB and BA domains is $\Delta V_{KP} \sim 60\,mV$ (Fig. S6), consistent with theoretical predictions of interfacial polarization (see Deb et al., Nature 2022, ref [10]). These values clearly demonstrate that the dominant contribution to the observed surface potential shift arises from the intrinsic interfacial polarization of the rhombohedral stacking, not from carrier density variations.

S9 Quantitative Work Function Analysis

To determine absolute work function values for different stacking configurations, we performed KPFM measurements on an additional sample containing monolayer MoS_2 and bilayer MoS_2 with AB and BA stacking domains on SiO_2 (Figure S7). Using the established monolayer MoS_2 work function (~4.7 eV, ref [26]), we observed minimal difference (<10 meV) between monolayer and bilayer AB configurations. The ~80 meV potential difference between AB and BA domains closely reproduces our original sample results (~60 meV), confirming consistent stacking-dependent charge transfer. These measurements yielded absolute work functions of 4.65 eV (AB) and 4.71 eV (BA) for bottom layers adjacent to graphene in the main original sample, both larger than the work function of the trilayer graphene substrate (~4.4 eV, ref [28]). This quantitatively validates the charge transfer mechanism underlying our optical detection method.

Reference work functions:

$$\phi_{3L\,Gr} \sim 4.4\,eV$$

$$\phi_{1L\,MoS_2} \sim 4.7\,eV$$

From the additional KPFM measurement (Figure S7):

$$\phi_{2L\,AB\,top} \sim \phi_{1L} + 10\,meV = 4.71\,eV$$

From our original sample measurement (Figure 1 in the main text):

$$\Delta\phi_{2L\,interlayer} \sim 0.06\,eV$$

Calculated absolute work function values for the main sample configuration:

$$\phi_{2L\,AB\,bottom} \sim 4.65\,eV$$

$$\phi_{2L\,BA\,bottom} \sim 4.71\,eV$$

$$\Delta\phi_{AB\rightarrow Gr} \sim 0.21\,eV < 0.27\,eV \sim \Delta\phi_{BA\rightarrow Gr}$$

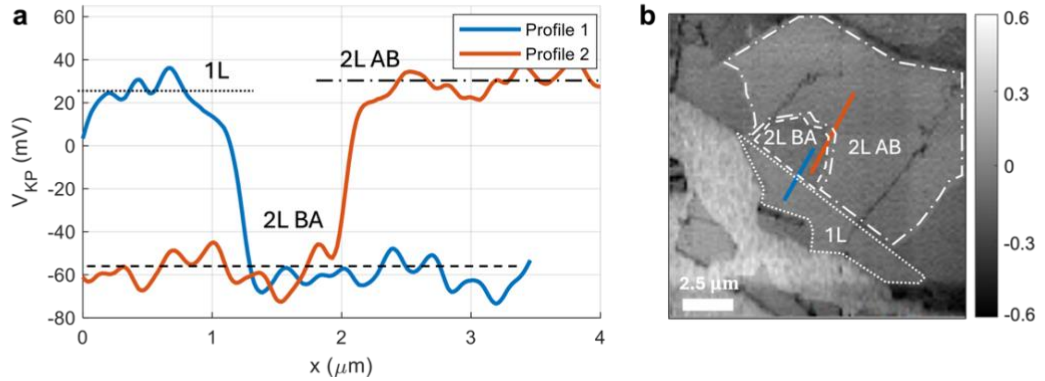

Figure S7: ***Surface potential of a monolayer–bilayer r-MoS_2 sample. a)*** *Line profiles of the surface potential (VKP) along the marked paths in b, showing distinct values for 1L, 2L AB, and 2L BA regions.* ***b)*** *KPFM map of the sample with domain annotations and profile locations.*

S10 References

S10.1 **Cheiwchanchamnangij, Tawinan; Lambrecht, Walter R. L.** “Quasiparticle Band Structure Calculation of Monolayer, Bilayer, and Bulk MoS_2.” *Physical Review B*, vol. 85, no. 20, 2 May 2012, article 205302. DOI: https://doi.org/10.1103/PhysRevB.85.205302.
